# Examining the role of lipids in hearing

**DOI:** 10.7554/eLife.111563

**Published:** 2026-05-13

**Authors:** Yein Christina Park, Angela Ballesteros

**Affiliations:** 1 Section on Sensory Physiology and Biophysics, National Institute on Deafness and other Communication Disorders Bethesda United States; 2 Graduate Program in Cell, Molecular, Developmental Biology and Biophysics, Johns Hopkins University Baltimore United States

**Keywords:** auditory hair cells, lipid bilayers, membrane asymmetry, scramblase activity, ATP8B1-TMEM30B, Mouse

## Abstract

The asymmetry of lipid membranes is tightly regulated in eukaryotic cells, and auditory hair cells are no exception.

**Related research article** De Hoyos HN, Li S, Im JS, Luz-Ricca A, Szeto B, Jonas R, Kim E, Amin N, Shin JB. 2026. ATP8B1-TMEM30B flippase activity maintains stereocilia lipid asymmetry required for hearing. *eLife* **15**:RP110466. doi: 10.7554/eLife.110466.

Why does our sense of hearing deteriorate with age or after repeated exposure to loud noise? Part of the answer to this question lies in the vulnerability of hair cells, the sensory cells in the inner ear that detect sound (Figure 1A). Humans are born with about 15,000 hair cells in each ear, and once they are lost, they are not replaced, so losing them can lead to permanent hearing loss (Wagner and Shin, 2019). But what determines whether the effect of stress on hair cells is reversible, such as the temporary ringing we experience in our ears after a loud concert, or if it will result in lasting hearing problems?

**Figure 1.**
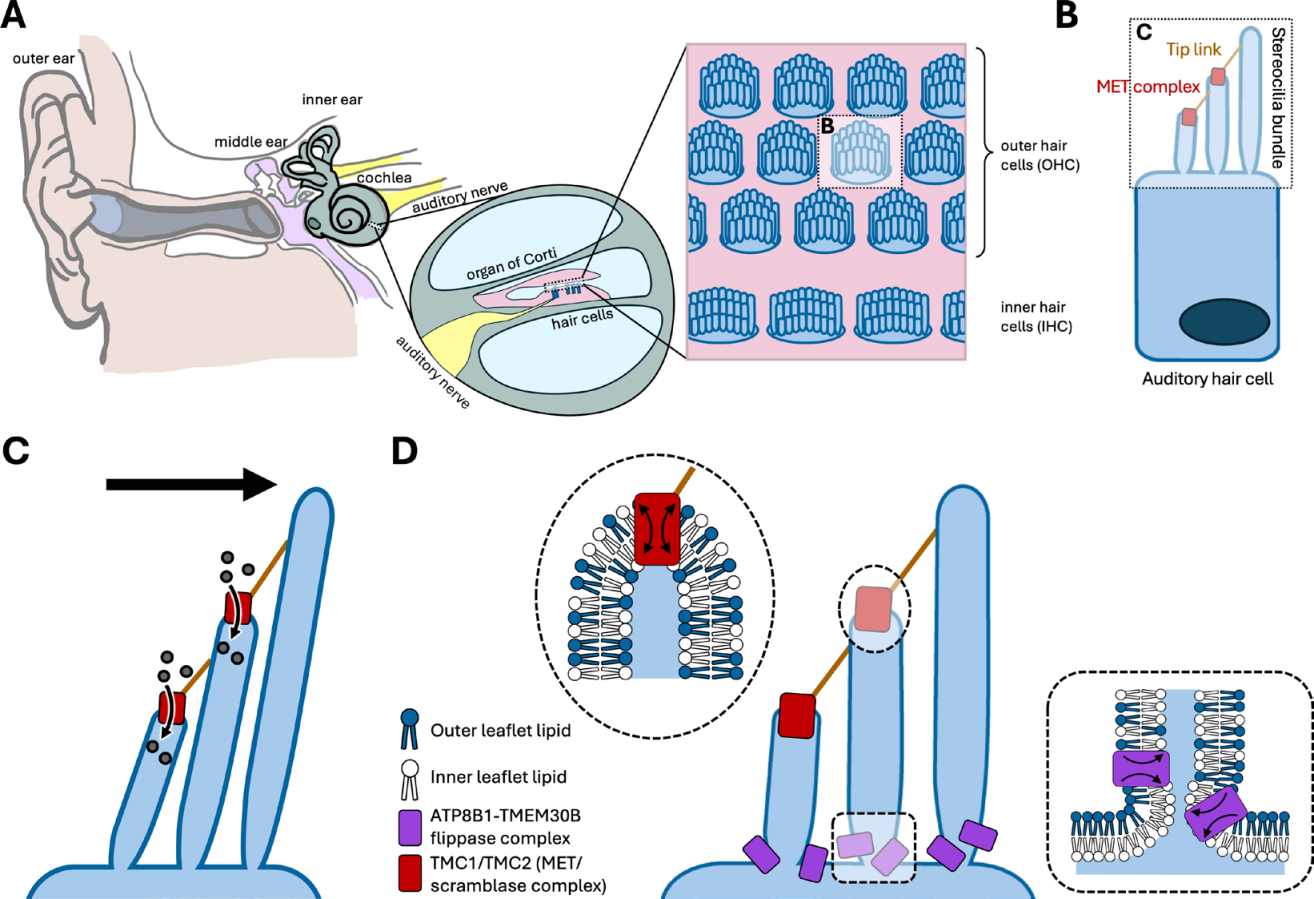
Regulation of membrane asymmetry in auditory hair cells. (**A**) Schematic illustration of the adult human ear, showing the cochlea (green), and the organ of Corti (pink). Within the organ of Corti are three rows of outer hair cells (blue) and one row of inner hair cells (also blue) which amplify sound and transmit signals to the auditory nerve (yellow), respectively. (**B**) Side view of an auditory hair cell, depicting the cylindrical organelles called stereocilia that project from its apical surface. The two shorter rows of stereocilia contain a mechanoelectrical transduction (MET) complex (red) at their tip. There are also protein links (brown) between the complexes on neighboring stereocilia. (**C**) When sound deflects the stereocilia, tension is applied to the MET complexes, causing an influx of ions (circles) into the hair cell. These ions produce the electrical signals that are sent from the auditory nerve (yellow) to the brain. (**D**) Three rows of stereocilia (center), and expanded views of the cell membrane at the stereocilia tip (left, oval) and base (right, rectangle). The cell membrane is made of two layers or leaflets of lipids (dark blue and white shapes), and in most cells the compositions of the inner and outer leaflets are different – a phenomenon known as membrane asymmetry. In hair cells, however, the scramblase activity of two proteins in the MET complex (red) – TMC1 and TMC2 – disrupt membrane asymmetry at the tip. De Hoyos et al. report that a ATP8B1-TMEM30B complex (purple) localizes at the base of the stereocilia, and that the flippase activity of this complex restores membrane asymmetry at the base by actively transporting specific lipids, such as phosphatidylserine and phosphatidylethanolamine (white shapes), to the inner leaflet.

An essential feature of hair cells – and, indeed, of most eukaryotic cells – is an uneven distribution of lipids between the inner and outer leaflets of the cell membrane. This membrane asymmetry – which is critical for many cellular functions (Sakuragi and Nagata, 2023) – is maintained by enzymes called flippases that move specific lipids to the inner leaflet (Bhandari et al., 2025), and floppases, which move lipids to the outer leaflet. There are also enzymes called scramblases that can move lipids in both directions, disrupting the membrane asymmetry. Importantly, scramblases are tightly regulated because they can move a lipid called phosphatidylserine to the outer leaflet, which marks the cell for elimination via the process of apoptotic cell removal. Remarkably, in hair cells, this scramblase activity happens within the very protein complex that detects sound.

This protein complex, known as the mechanoelectrical transduction complex, converts sound into electrical signals to enable hearing (Figure 1B, C). Existing evidence indicates that its core components, a pair of proteins called TMC1 and TMC2, function both as ion channels and lipid scramblases. As ion channels, they allow ions to enter the hair cells, initiating electrical signals that are sent to the brain; and as scramblases, they disrupt membrane asymmetry. One possible explanation for this dual function is that lipid reorganization by TMC1 and TMC2 alters membrane properties, thereby tuning the ion channel function of the complex (George and Ricci, 2026).

However, when left unchecked, scramblase activity can – via the movement of phosphatidylserine to the outer leaflet, and the formation of blebs on the membrane – lead to the death of hair cells in mutant TMC1 mouse models (Ballesteros and Swartz, 2022; Lee et al., 2025; Peineau et al., 2025). These findings suggest that hair cells must tightly regulate membrane asymmetry by balancing scramblase, flippase, and floppase activities to preserve hearing. Now, in eLife, Jung-Bum Shin and colleagues at the University of Virginia – including Henry De Hoyos as first author – report that they have identified some of the key players involved in the regulation of membrane asymmetry in hair cells (De Hoyos et al., 2026).

De Hoyos et al. identified an ATPase called ATP8B1 as the predominant flippase in outer hair cells and determined its timeline of expression in mice. ATP8B1 protein expression is first detected in hair cells around postnatal day 11, coinciding with the onset of hearing, suggesting a potential role in auditory maturation. They also showed that ATP8B1 and its chaperone – a transmembrane protein called TMEM30B – were localized to the bases of stereocilia, which are cylindrical structures erected at the top of the hair cells (Figure 1B). This finding supports a model in which the scramblase activity of TMC1 and TMC2 at the tips of the stereocilia is counterbalanced by the flippase activity of the ATP8B1–TMEM30B complex at the base, thus limiting the diffusion of externalized phosphatidylserine (Figure 1D).

Building on these findings, De Hoyos et al. examined how the loss of ATP8B1 or TMEM30B affects outer hair cells. In mice lacking either protein, outer hair cells displayed disrupted membrane asymmetry, including phosphatidylserine externalization, stereocilia degeneration, and cell death. These defects were accompanied by significant hearing impairment compared to controls. Notably, when TMEM30B was absent, ATP8B1 mislocalized to the hair cell bodies, demostrating that proper localization of ATP8B1 is essential for the survival of outer hair cells and hearing. Finally, De Hoyos et al. found that in mutant mice lacking the gene for Cib2 – which encodes an essential component of the mechanoelectrical transduction complex (Giese et al., 2017; Liang et al., 2021) – ATP8B1 and TMEM30B were mislocalized to the body of the hair cells. This suggests that a working mechanoelectrical transduction complex is required for correct flippase localization.

This work marks an important step toward understanding the role of lipid flippases in hearing, and adds to growing evidence that membrane asymmetry is closely linked to the activity of the mechanoelectrical transduction complex and hair cell health. Although the details of the underlying mechanism remain unclear, it is evident that the activity of the complex – which is composed of multiple transmembrane proteins – simultaneously modulates, and is modulated by, its lipid environment. This regulation can be direct (through the scramblase activity of TMC1 and TMC2, or interactions between the complex and a lipid called PIP2; Caprara et al., 2025; Cunningham et al., 2020; Effertz et al., 2017), or indirect, through changes in the localization of other membrane regulatory proteins, such as ATP8B1 and TMEM30B (De Hoyos et al., 2026).

Despite these advances, however, key questions remain. For example, it remains unclear whether additional flippases are localized near the mechanoelectrical transduction complex. The identity of the flippases that establish membrane asymmetry in inner hair cells are also unknown, and the role of floppases remains unexplored. Moreover, a particular mutation in ATP8B1 (called G308V) causes hearing loss in mice (Stapelbroek et al., 2009), but it is not clear if this reflects ATP8B1 G308V mislocalization or changes to the mechanoelectrical transduction complex. Addressing these gaps in knowledge will be critical for understanding how membrane asymmetry supports hair cell function and preserves hearing.

**Acknowledgement**: This work was supported by the Division of Intramural Research of the National Institute on Deafness and Other Communication Disorders (NIDCD DIR DC000096 to AB) and by the Intramural Research Program of the National Institutes of Health (NIH). The contributions of the NIH author are considered Works of the United States Government. The findings and conclusions are those of the authors and do not necessarily reflect the views of the NIH or the US Department of Health and Human Services.

## References

[bib1] Ballesteros A, Swartz KJ (2022). Regulation of membrane homeostasis by TMC1 mechanoelectrical transduction channels is essential for hearing. Science Advances.

[bib2] Bhandari N, Prince A, Khan MR, Traughber CA, Neupane K, Lorkowski SW, Brubaker G, Ertugral EG, Kothapalli CR, Dubyak GR, Smith JD, Gulshan K (2025). ATP8B1 regulates PIP2 localization and cleavage of pyroptotic executioner Gasdermin D. PNAS.

[bib3] Caprara GA, Kim Y, Jun S, Li S, Kim U, Shin JB, Peng AW (2025). PIP_2_-Tmie interactions drive mammalian hair cell slow adaptation independently of myosin motors. bioRxiv.

[bib4] Cunningham CL, Qiu X, Wu Z, Zhao B, Peng G, Kim YH, Lauer A, Müller U (2020). TMIE defines pore and gating properties of the mechanotransduction channel of mammalian cochlear hair cells. Neuron.

[bib5] De Hoyos HN, Li S, Im JS, Luz-Ricca A, Szeto B, Jonas R, Kim E, Amin N, Shin JB (2026). ATP8B1-TMEM30B flippase activity maintains stereocilia lipid asymmetry required for hearing. eLife.

[bib6] Effertz T, Becker L, Peng AW, Ricci AJ (2017). Phosphoinositol-4,5-bisphosphate regulates auditory hair-cell mechanotransduction-channel pore properties and fast adaptation. Journal of Neuroscience.

[bib7] George SS, Ricci AJ (2026). Auditory hair cell mechanotransduction channels dynamically shape the mechanical properties of their membrane environment. Advanced Science.

[bib8] Giese APJ, Tang YQ, Sinha GP, Bowl MR, Goldring AC, Parker A, Freeman MJ, Brown SDM, Riazuddin S, Fettiplace R, Schafer WR, Frolenkov GI, Ahmed ZM (2017). CIB2 interacts with TMC1 and TMC2 and is essential for mechanotransduction in auditory hair cells. Nature Communications.

[bib9] Lee H, Park YC, Wen H, Smith HE, Balaraman J, Cui R, Sotomayor M, Ballesteros A (2025). TMC1 and TMC2 are cholesterol-dependent scramblases that regulate membrane homeostasis in auditory hair cells. bioRxiv.

[bib10] Liang X, Qiu X, Dionne G, Cunningham CL, Pucak ML, Peng G, Kim YH, Lauer A, Shapiro L, Müller U (2021). CIB2 and CIB3 are auxiliary subunits of the mechanotransduction channel of hair cells. Neuron.

[bib11] Peineau T, Marcovich I, Rodriguez C, O’Malley S, Cui R, Ballesteros A, Holt JR (2025). Mammalian TMC1 or 2 are necessary for scramblase activity in auditory hair cells. Hearing Research.

[bib12] Sakuragi T, Nagata S (2023). Regulation of phospholipid distribution in the lipid bilayer by flippases and scramblases. Nature Reviews Molecular Cell Biology.

[bib13] Stapelbroek JM, Peters TA, van Beurden DHA, Curfs JHAJ, Joosten A, Beynon AJ, van Leeuwen BM, van der Velden LM, Bull L, Oude Elferink RP, van Zanten BA, Klomp LWJ, Houwen RHJ (2009). ATP8B1 is essential for maintaining normal hearing. PNAS.

[bib14] Wagner EL, Shin JB (2019). Mechanisms of hair cell damage and repair. Trends in Neurosciences.

